# Supplementary material for: Acacetin Inhibits the Growth of STAT3-Activated DU145 Prostate Cancer Cells by Directly Binding to Signal Transducer and Activator of Transcription 3 (STAT3)
Source: Molecules. 2021 Oct 14;26(20):6204. doi: 10.3390/molecules26206204 (PMC8540902; doi:10.3390/molecules26206204)
Supplement: Supplementary file 1 [file molecules-26-06204-s001.zip › Molecules-1382193-Supplementary.pdf]

molecules

MDPI

Supplementary Material

Acacetin Inhibits the Growth of STAT3-Activated DU145 Prostate Cancer Cells by Directly Binding to Signal Transducer and Activator of Transcription 3 (STAT3)

Sun Yun, Yu-Jin Lee, Jiyeon Choi, Nam Doo Kim, Dong Cho Han and Byoung-Mog Kwon

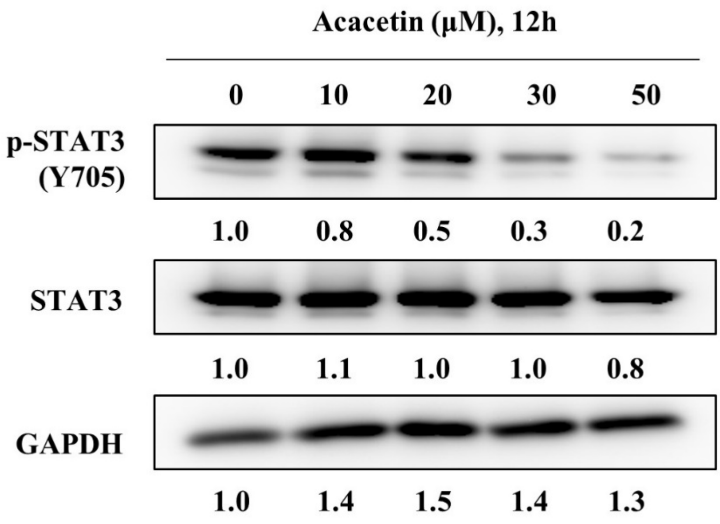

Figure S1. Acacetin inhibits p-STAT3-Y705 in HCC827. HCC827 cells were treated with acacetin for 12 h; whole lysate was obtained and analyzed by western blotting with p-STAT3 (Y705) and STAT3 antibodies. The band intensity of each protein was quantified using the Multi Gauge program.

molecules
MDPI

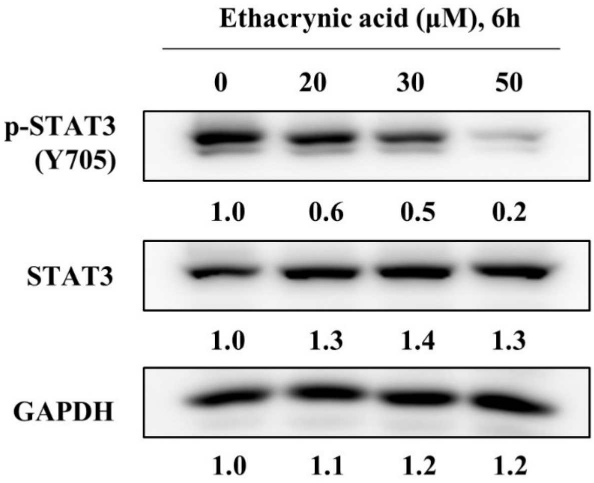

Figure S2. Ethacrynic acid inhibits p-STAT3-Y705 in DU145. DU145 cells were treated with ethacrynic acid for 6 h; whole lysate was obtained and analyzed by western blotting with p-STAT3 (Y705) and STAT3 antibodies. The band intensity of each protein was quantified using the Multi Gauge program
